# A Competing-Risk Approach for Modeling Length of Stay in Severe Malaria Patients in South-East Asia and the Implications for Planning of Hospital Services

**DOI:** 10.1093/cid/ciy211

**Published:** 2018-03-19

**Authors:** Claire M Keene, Arjen Dondorp, Jane Crawley, Eric O Ohuma, Mavuto Mukaka

**Affiliations:** 1 Centre for Tropical Medicine & Global Health, Nuffield Department of Medicine, University of Oxford, Peter Medawar Building for Pathogen Research, United Kingdom; 2 Mahidol Oxford Tropical Medicine Research Unit, Rajthevee, Bangkok, Thailand; 3 Centre for Statistics in Medicine, University of Oxford, Botnar Research Centre, United Kingdom

**Keywords:** length of stay, time to death, time to discharge, competing risk, severe malaria

## Abstract

**Background:**

Management of severe malaria with limited resources requires comprehensive planning. Expected length of stay (LOS) and the factors influencing it are useful in the planning and optimisation of service delivery.

**Methods:**

A secondary, competing-risk approach to survival analysis was performed for 1217 adult severe malaria patients from the South-East Asia Quinine Artesunate Malaria Trial.

**Results:**

Twenty percent of patients died; 95.4% within 7 days compared to 70.3% of those who were discharged. Median time to discharge was 6 days. Compared to quinine, artesunate increased discharge incidence (subdistribution-Hazard ratio, 1.24; [95% confidence interval 1.09–1.40]; *P* = .001) and decreased incidence of death (0.60; [0.46–0.80]; *P* < .001). Low Glasgow coma scale (discharge, 1.08 [1.06–1.11], *P* < .001; death, 0.85 [0.82–0.89], *P* < .001), high blood urea-nitrogen (discharge, 0.99 [0.99–0.995], *P* < .001; death, 1.00 [1.00–1.01], *P* = .012), acidotic base-excess (discharge, 1.05 [1.03–1.06], *P* < .001; death, 0.90 [0.88–0.93], *P* < .001), and development of shock (discharge, 0.25 [0.13–0.47], *P* < .001; death, 2.14 [1.46–3.12], *P* < .001), or coma (discharge, 0.46 [0.32–0.65], *P* < .001; death, 2.30 [1.58–3.36], *P* < .001) decreased cumulative incidence of discharge and increased incidence of death. Conventional Kaplan-Meier survival analysis overestimated cumulative incidence compared to competing-risk model.

**Conclusions:**

Clinical factors on admission and during hospitalisation influence LOS in severe malaria, presenting targets to improve health and service efficiency. Artesunate has the potential to increase LOS, which should be accounted for when planning services. In-hospital death is a competing risk for discharge; an important consideration in LOS models to reduce overestimation of risk and misrepresentation of associations.

## Teaser

Conventional survival analysis overestimated cumulative incidence of death and discharge, whereas competing-risk approaches gave more precise estimates and relevant insights on predictive factors in severe malaria, revealing that artesunate increased overall length of stay, an important consideration for planning services.

Malaria remains a prominent global health issue, with 3.2 billion people at risk of death and disability worldwide [1, 2], mostly in areas with fewer resources to manage it [3]. It is responsible for up to 50% of all hospital admissions in endemic areas [4], encumbering healthcare systems with an estimated global case-management cost of $300 million per year [5].

With finite resources available, planning efficient service delivery requires careful resource allocation, informed by anticipated hospital bed requirements and expected resource use [6, 7]. This is predicted using estimated length of stay (LOS) [8, 9], a metric widely used in hospital capacity planning [10] and recommended by the World Health Organization (WHO) to indicate health service quality and efficiency [11].

Longer LOS increases resource consumption [12], so targeting modifiable factors that influence LOS can improve health system efficiency and reduce expenditure [13]. However, there is little information on factors that affect LOS in severe malaria, the pathway to death in malaria patients [14]. Unsurprisingly, severe malaria is more expensive to treat than uncomplicated malaria, primarily driven by differences in LOS [15]. Yet, most studies focus on predictors of mortality [16], resulting in insufficient evidence on LOS in severe malaria to make informed decisions.

Treatment consumes more than a third of the global malaria budget [17], making it an important policy consideration. Intravenous artesunate is superior to quinine; it reduced mortality by 34.7% in Asia [18] and 22.5% in African children [19], clears parasites faster, causes fewer adverse effects, and is easier to administer [18]. WHO has since endorsed artesunate as first-line treatment for severe malaria [20], but by 2016, less than 65% of countries had adopted this recommendation into policy [3]. Understanding artesunate’s impact on LOS could contribute to these policy decisions and aid service-delivery planning.

Methods for estimating LOS conventionally analyze time to discharge; time to death in-hospital does not contribute to LOS estimates. Instead, deaths are censored in a standard survival analysis approach [21], ignoring the influence that time to death has on resource use and the impact deaths have on the cumulative incidence of discharge. The complement of the Kaplan-Meier survival curve is often used to estimate cumulative incidence, assuming that the probability of the primary event is the same in the censored individuals as in those still under observation (ie, censoring events are random) [22]. When a patient dies in-hospital, discharge is no longer possible, so censoring the competing event of death violates this assumption and leads to overestimation of discharge incidence in a Kaplan-Meier model [23]. Death and discharge “compete” with each other, necessitating the use of a competing-risk approach that generates results that reflect real-world situations where competing events are present [24]. Because more accurate information means better informed decisions on resource allocation [25], we modeled LOS in severe malaria accounting for the competing event of death.

## METHODS

This was a retrospective, secondary analysis of the South-East Asian Quinine Artesunate Malaria Trial (SEAQUAMAT) dataset, modeling LOS as time to discharge or death in-hospital, with competing-risk methodology.

### Data

**Figure 1.**
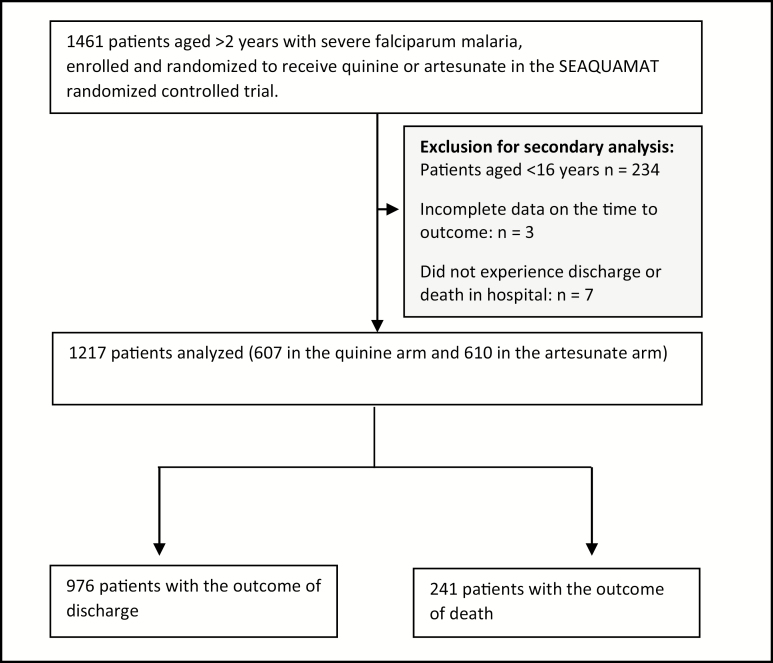
Eligibility flow chart for the secondary analysis of the South-East Asian Quinine Artesunate Malaria Trial dataset. Abbreviation: SEAQUAMAT, South-East Asian Quinine Artesunate Malaria Trial.

SEAQUAMAT was a randomized, controlled trial that found that artesunate decreased death by 34.7% compared to quinine [18]. Patients from Myanmar, Bangladesh, India, and Indonesia (where 97% of the confirmed malaria cases in the Asia-Pacific region occur [26]) with severe falciparum malaria (diagnosed by clinical criteria and a positive rapid test) were enrolled between June 2003 and May 2005 [18]. In this analysis, 234 children aged <16 years were excluded to prevent confounding of associations with variables that have age-dependent normal ranges. Seven patients refused treatment and died at home; they did not experience either event of interest (discharge or death in-hospital) and were excluded from this analysis in addition to patients who had incomplete data on time to outcome. Ultimately, we analyzed 1217 patients (Figure 1).

### Statistical Methods

A literature search using the Cochrane Library, PubMed, and Google Scholar databases up to 1 August 2017 determined factors associated with malaria LOS (Supplementary Tables 1 and 2). Objective measures were chosen to represent clinical factors where available (eg, blood urea nitrogen [BUN] representing renal function); base-excess was chosen over pH as a better measure of metabolic acidosis [27]. Complications that develop during hospitalization, not reported in the literature but available in the dataset, were explored for their potential influence on LOS.

After discussion with clinicians, 19 variables were chosen for investigation: demographic factors (country, age, and gender), clinical factors on admission (systolic blood pressure, respiratory rate, temperature, Glasgow coma scale [GCS], and seizures), admission laboratory results (BUN, base-excess, parasite count, and hemoglobin), clinical conditions that developed during hospitalization (shock, coma, seizures, sepsis, and anemia), and treatment (previous antimalarial treatment and treatment with artesunate or the control, quinine).

Normally distributed variables were summarized with means and standard deviations. Non-normally distributed variables were summarized with medians and interquartile ranges (IQRs) or log-transformed to ensure a normal distribution (eg, parasite count). χ^2^ tests were used to examine categorical variable associations. Half a day was added to the LOS of patients discharged on the same day as admission rather than excluding them from analysis, as it was assumed that time must pass before an event could be observed.

Cumulative incidence illustrates the pattern of events over time and has been suggested as more relevant for planning purposes than the event rate alone [28]. Cumulative incidence is traditionally represented by the complement of the Kaplan-Meier curve, and this was calculated and compared to a competing-risk model of cumulative incidence.

Associations were examined with cause-specific hazard (CSH) analysis using Cox proportional hazards regression [22, 24], describing the hypothetical probability of an event in a world where other events do not exist and quantifying a variable’s effect on the hazard (rate) of an outcome (cause of failure) [28]. Subdistribution-Hazard (SDH) ratios, estimated with a Fine and Gray model, demonstrate associations with cumulative incidence accounting for competing risks [29], where a ratio greater than 1 indicates a positive effect.

Univariable CSH and SDH analyses were performed for each outcome (Supplementary Tables 3 and 4). Factors with a significant effect (*P* < .05) were assessed in a multivariable model for discharge and death separately. Treatment (the randomized variable) and country were adjusted for as fixed effects throughout all models.

Analysis was performed using Stata, version 12, statistical software package. Confidence intervals (CIs) were reported as 95%, and the threshold for significance was *P* < .05. Findings were reported according to the STROBE (Strengthening the Reporting of Observational Studies in Epidemiology) Statement Cohort Studies checklist [30].

Local ethics committees and the Oxford Tropical Research Ethics Committee approved the original study. Written, informed consent was provided by patients, or their attendant relative, on enrollment into the original study [18], and there was no further recruitment of participants for this analysis. Permission to use the data was obtained from the Mahidol-Oxford Research Unit.

## RESULTS

Of the 1217 patients analyzed, 976 (80.2%) were discharged and 241 (19.8%) died in-hospital. Most patients were male (76.0%) and recruited from Myanmar (38.3%); the median age was 28 years (range, 16–87 years). A total of 235 patients (19.3%) developed 1 or more complications while admitted in hospital (Table 1). The median LOS (death or discharge) was 5 days (IQR 3–7, range 0.4–54 days).

Those who died were older than those who survived (median age 30 vs 27 years), with a lower GCS (median score 8 vs 13), worse renal function (median BUN 71 mg/dL vs 35 mg/dL), more severe acidosis (mean base-excess −11.42 vs −2.83), a higher parasite count (geometric mean 81737 vs 31983 parasites/µL), developed more clinical complications during admission (49.8% vs 11.8%), and were more likely to receive quinine (61.0% vs 39.0% receiving artesunate; Table 1).

**Table 1.** Baseline Characteristics of the Adult Population of the South-East Asian Quinine Artesunate Malaria Trial Dataset

Variable	Whole Sample	Discharged	Died	*P* Value
**Demographics**				
Country (total n)	1217	976	241	**<.001**
Myanmar n (%)	466 (38.3)	395 (40.5)	71 (29.5)	
Bangladesh n (%)	418 (34.4)	302 (30.9)	116 (48.1)	
India n (%)	137 (11.3)	105 (10.8)	32 (13.3)	
Indonesia n (%)	196 (16.1)	174 (17.8)	22 (9.1)	
Age in years (total n)	1217	976	241	**<.001**
Median and IQR	28 (21;40)	27 (21;38)	30 (23;45)	
Gender (total n)	1217	976	241	.835
Females (reference) n (%)	292 (24.0)	234 (24.0)	58 (24.1)	
Males n (%)	925 (76.0)	742 (76.0)	183 (75.9)	
**Clinical factors on admission**				
Systolic blood pressure in mmHg (total n)	1207	971	236	.224
Median and IQR	100 (90;120)	100 (90;120)	110 (92;120)	
Respiratory rate in breaths/minute (total n)	1215	975	240	**<.001**
Median and IQR	24 (20;32)	24 (20;32)	28 (22;36)	
Temperature in C (total n)	1215	975	240	**.023**
Mean and SD	38.05 (1.18)	38.09 (1.19)	37.90 (1.12)	
Glasgow Coma Scale score (total n)	1217	976	241	**<.001**
Median and IQR	12 (9;15)	13 (9.5;15)	8 (6;11)	
Seizures (total n)	1217	976	241	.258
Seizures present n (%)	114 (9.4)	85 (8.7)	29 (12.0)	
**Laboratory results on admission**				
Blood urea nitrogen mg/dL (total n)	1157	933	224	**<.001**
Median and IQR	30 (17;60)	35 (15;45)	71 (37.5;107.5)	
Base-excess mmol/L (total n)	1103	890	213	**<.001**
Mean and SD	-4.49 (6.95)	-2.83 (5.53)	-11.42 (7.93)	
Log parasite count (total n)	1153	921	232	**<.001**
Mean and SD	10.56 (2.41)	10.37 (2.38)	11.31 (2.38)	
Haemoglobin mg/dL(total n)	1142	920	222	.631
Mean and SD	10.19 (3.31)	10.21 (3.33)	10.09 (3.24)	
**Clinical conditions developed during admission**				
Shock noted (total n)	1217	976	241	**<.001**
Shock developed n (%)	54 (4.4)	11 (1.1)	43 (17.8)	
Coma noted (total n)	1217	976	241	**<.001**
Coma developed n (%)	103 (8.5)	43 (4.4)	60 (24.9)	
Seizures noted (total n)	1217	976	241	**<.001**
Seizures developed n (%)	58 (4.7)	23 (2.4)	35 (14.5)	
Sepsis noted (total n)	1217	976	241	**<.001**
Sepsis developed n (%)	85 (7.0)	49 (5.0)	36 (14.9)	
Anaemia noted (total n)	1217	976	241	.911
Anaemia developed n (%)	29 (2.4)	23 (2.4)	6 (2.5)	
**Treatment**				
Previous effective antimalarials (total n)	1217	976	241	.675
Previous treatment given n (%)	216 (17.8)	171 (17.5)	45 (18.7)	
Study treatment (total n)	1217	976	241	**.001**
Quinine (reference) n (%)	607 (49.9)	460 (47.1)	147 (61.0)	
Artesunate n (%)	610 (50.1)	516 (52.9)	94 (39.0)	

The bold values are those that are significant results (ie, *P* < .05).

Abbreviations: IQR, inter-quartile range; SD, standard deviation.

### Time to Discharge

**Figure 2.**
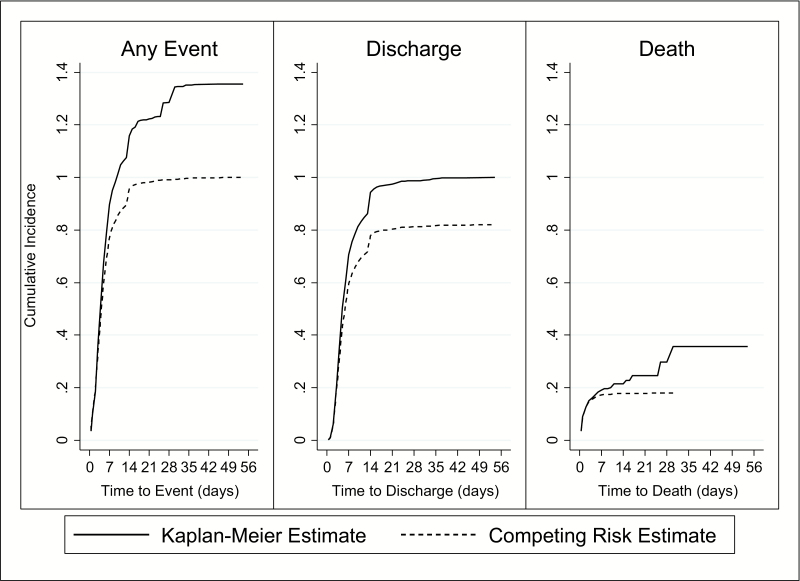
Cumulative incidence of any event (discharge or death in-hospital), discharge (accounting for death), and death (accounting for discharge), estimated using Kaplan-Meier and competing-risks methods.

**Figure 3.**
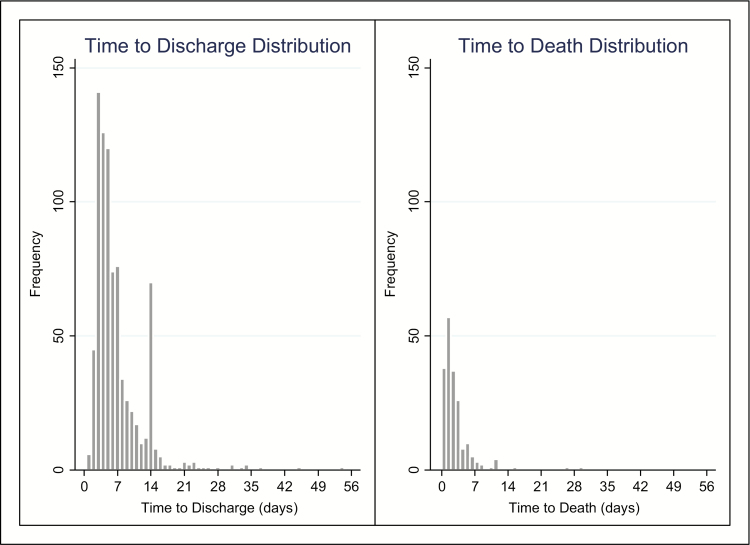
Distribution of time to discharge and time to death, in days.

The median time to discharge was 6 days (IQR 4–9, range 0.5–54 days), with 80% of the patients cumulatively discharged (Figure 2). The Kaplan-Meier model alternatively estimated cumulative discharge incidence as 100%. Time to discharge had a right skewed distribution, with most patients discharged on day 3 (16.1%) and most discharged in the first week (70.2%), with a secondary peak at 14 days (8.8%; Figure 3).

The adjusted rate of discharge was increased 5% for every unit increase in the GCS score (cause-specific hazard ratio [CSHR] 1.05; [1.02–1.07]; *P* < .001). It was decreased 1% for every unit increase in BUN (CSHR: 0.99; [0.99–0.996]; *P* < .001), 31% by development of coma (CSHR: 0.69; [0.49–0.97]; *P* = .034), 40% by development of seizures (CSHR: 0.60; [0.38–0.93]; *P* = .024), and 55% with development of sepsis (CSHR: 0.45; [0.33–0.63]; *P* =< 0.001; Table 2).

**Table 2.** Multivariable Analysis for Time to Discharge, Using a Conventional Cox Regression Model to Obtain a Cause-specific Hazard Ratio, and a Fine and Gray Competing-risks Method to Obtain a Subdistribution-Hazard Ratio

Covariate	Cause-specific Hazard (Rate of Discharge)		Subdistribution-Hazard (Association With Cumulative Incidence of Discharge)	
	CSH Ratio	*P* Value	SDH Ratio	*P* Value
**Demographics**				
Country				
Myanmar (reference)				
Bangladesh	1.95 (1.61–2.34)	**<.001**	1.58 (1.33–1.88)	**<.001**
India	2.17 (1.69–2.79)	**<.001**	2.35 (1.92–2.86)	**<.001**
Indonesia	3.29 (2.64–4.10)	**<.001**	2.80 (2.27–3.45)	**<.001**
Age	NS		1.00 (0.99–1.00)	.270
**Clinical factors on admission**				
Systolic blood pressure	NS		0.99 (0.99–0.997)	**<.001**
Respiratory rate	NS		1.01 (1.00–1.02)	**.034**
Temperature	1.07 (1.01–1.14)	**.022**	1.12 (1.05–1.17)	**<.001**
Glasgow coma scale	1.05 (1.02–1.07)	**<.001**	1.08 (1.06–1.11)	**<.001**
Seizures	NS		0.98 (0.77–1.24)	.876
**Laboratory results on admission**				
Blood urea nitrogen	0.99 (0.99–0.996)	**<.001**	0.99 (0.99–0.995)	**<.001**
Base-excess	1.01 (1.00–1.03)	.077	1.05 (1.03–1.06)	**<.001**
Log parasite count	0.98 (0.95–1.01)	.147	0.98 (0.95–1.01)	.183
Haemoglobin	1.02 (1.00–1.04)	.123	NS	
**Clinical conditions developed during admission**				
Shock developed	0.59 (0.30–1.14)	.116	0.25 (0.13–0.47)	**<.001**
Coma developed	0.69 (0.49–0.97)	**.034**	0.46 (0.32–0.65)	**<.001**
Seizures developed	0.60 (0.38–0.93)	**.024**	0.55 (0.38–0.81)	**.002**
Sepsis developed	0.45 (0.33–0.63)	**<.001**	0.46 (0.32–0.65)	**<.001**
**Treatment**				
Quinine (reference)	NS but adjusted for in the multivariable model			
Artesunate			1.24 (1.09–1.40)	**.001**

The bold values are those that are significant results (ie, *P* < .05).

Abbreviations: CSH, cause-specific hazard; NS, non-significant on univariable analysis and thus not included in the multivariable model; SDH, subdistribution hazard.

Cumulative incidence of discharge was increased 8% for every unit increase in GCS score (subdistribution-Hazard ratio [SDHR], 1.08; [1.06–1.11]; *P* < .001) and 5% for every unit increase in base-excess (SDHR: 1.05; [1.03–1.06]; *P* < .001). It was decreased 1% per unit increase in BUN (SDHR: 0.99; [0.99–0.995]; *P* < .001), 75% with development of shock (SDHR: 0.25; [0.13–0.47]; *P* < .001), 54% by development of coma (SDHR: 0.46; [0.32–0.65]; *P* < .001), 45% by development of seizures (SDHR: 0.55; [0.38–0.81]; *P* = .002), and 54% by development of sepsis (SDHR: 0.46; [0.32–0.65]; *P* < .001; Table 2).

Median time to discharge was 5 days for patients with a normal GCS (score of 15), 6 days for those with a low score (GCS 8–14), and 7 days in those with coma on presentation (GCS <8). Those who developed shock or seizures each had a median time to discharge of 14 days, those who developed sepsis had 11 days and those who developed a coma during hospitalisation had a median time to discharge of 10 days. Most who developed complications (81.8% for shock, 67.4% for coma, 78.3% for seizures, and 59.2% for sepsis) were discharged after 7 days.

**Figure 4.**
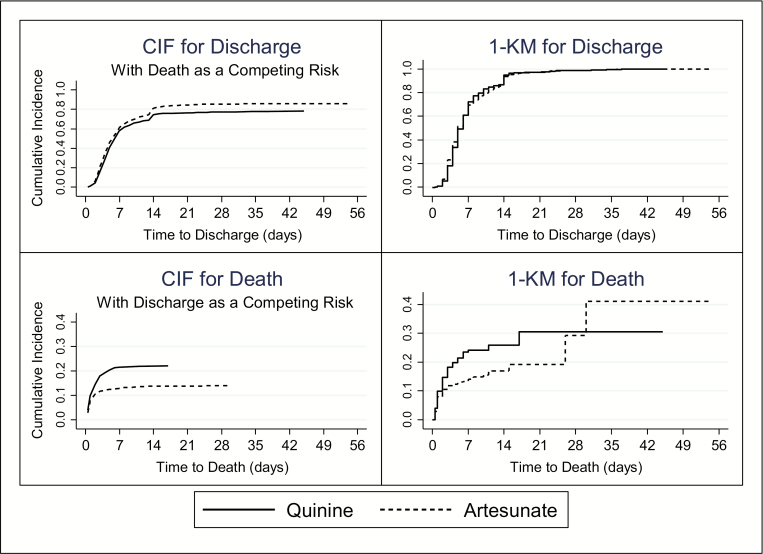
Cumulative incidence of discharge and death in-hospital by treatment arm (quinine or artesunate), estimated with competing-risks and Kaplan-Meier models. Abbreviations: CIF, competing-risks; 1-KM, Kaplan-Meier.

The median time to discharge was 6 days for both treatments, ranging from 0.5 to 54 days for artesunate and 1 to 45 days for quinine. There was no difference in proportions of patients discharged after 7 days in the artesunate (30.8%) and quinine (28.7%) arms (*P* = .470). Treatment was not associated with the rate of discharge (CSHR: 1.08; [0.94–1.25]; *P* = .253), but artesunate did increase cumulative incidence of discharge (SDHR: 1.24; [1.09–1.40]; *P* = .001; Table 2), resulting in 85.0% of artesunate recipients ultimately discharged compared to 76.0% for quinine recipients (Figure 4).

### Time to Death

The cumulative incidence of death was 20%; alternatively estimated as 40% using the Kaplan-Meier approach (Figure 2). The mean time to death was 2.5 days (standard deviation, 3.4 days), ranging from 0.5 to 30 days. Time to death was right skewed, with most patients dying on the first day after admission (31.1%) and 95.4% dying in the first week (Figure 3).

The adjusted rate of death at any point was decreased 14% for every unit increase in GCS score (CSHR: 0.86; [0.82–0.90]; *P* < .001) and 10% per unit increase in base-excess (CSHR: 0.90; [0.88–0.92]; *P* < .001). Development of shock increased the adjusted rate of death 2.11 times ([1.38–3.21]; *P* = .001) and development of coma 2.28 times ([1.53–3.40]; *P* < .001; Table 3).

The cumulative incidence of death was decreased 15% for every unit increase in GCS score (SDHR: 0.85; [0.82–0.89]; *P* < .001) and 10% per unit increase in base-excess (SDHR: 0.90; [0.88–0.93]; *P* < .001). Development of shock increased the cumulative incidence of death 2.14 times ([1.46–3.12]; *P* < .001) and development of coma 2.30 times ([1.58–3.36]; *P* < .001; Table 3). Significant variables predictive of each outcome are summarized in Table 4.

**Table 3.** Multivariable Analysis for Time to Death, Using a Conventional Cox Regression Model to Obtain a Cause-specific Hazard Ratio, and a Fine and Gray Competing-risks Method to Obtain a Subdistribution-Hazard Ratio

Covariate	Cause-specific Hazard (Rate of Death)		Subdistribution-Hazard (Association With Cumulative Incidence of Death)	
	CSH Ratio	*P* Value	SDH Ratio	*P* Value
**Demographics**				
Country				
Myanmar (reference)				
Bangladesh	1.72 (1.15–2.57)	**.008**	1.68 (1.15–2.45)	**.007**
India	0.69 (0.41–1.17)	.165	0.66 (0.41–1.05)	.081
Indonesia	0.98 (0.55–1.74)	.941	0.91 (0.56–1.47)	.7691
Age	1.01 (1.00–1.02)	**.044**	1.01 (1.00–1.02)	**.044**
**Clinical factors on admission**				
Systolic blood pressure	1.01 (1.00–1.02)	**.004**	1.01 (1.00–1.02)	**.001**
Respiratory rate	1.01 (0.99–1.02)	.404	1.01 (0.99–1.02)	.318
Temperature	0.86 (0.74–1.00)	**.047**	0.84 (0.73–0.97)	**.021**
Glasgow coma scale	0.86 (0.82–0.90)	**<.001**	0.85 (0.82–0.89)	**<.001**
**Laboratory results on admission**				
Blood urea nitrogen	1.00 (1.00–1.01)	**.038**	1.00 (1.00–1.01)	**.012**
Base-excess	0.90 (0.88–0.92)	**<.001**	0.90 (0.88–0.93)	**<.001**
Log parasite count	1.07 (1.00–1.15)	.061	1.08 (1.00–1.17)	.059
**Clinical conditions developed during admission**				
Shock developed	2.11 (1.38–3.21)	**.001**	2.14 (1.46–3.12)	**<.001**
Coma developed	2.28 (1.53–3.40)	**<.001**	2.30 (1.58–3.36)	**<.001**
Seizures developed	1.40 (0.91–2.16)	.121	1.45 (0.98–2.15)	.065
Sepsis developed	0.99 (0.63–1.55)	.964	1.06 (0.68–1.64)	.802
**Treatment**				
Quinine (reference)				
Artesunate	0.60 (0.45–0.80)	**.001**	0.60 (0.46–0.80)	**<.001**

The bold values are those that are significant results (ie, *P* < .05).

Abbreviations: CSH, cause-specific hazard; SDH, subdistribution hazard.

**Table 4.** Significant Predictive Variables in Each of the Multivariable Cause-Specific and Subdistribution-Hazard Models for Each Event

CSH for Discharge	SDH for Discharge	CSH for Death	SDH for Death
Country	Country	Country	Country
Temperature	Blood pressure	Age	Age
Glasgow coma scale	Respiratory rate	Blood pressure	Blood pressure
Blood urea nitrogen	Temperature	Temperature	Temperature
Coma developed	Glasgow coma scale	Glasgow coma scale	Glasgow coma scale
Seizures developed	Blood urea nitrogen	Blood urea nitrogen	Blood urea nitrogen
Sepsis developed	Base-excess	Base-excess	Base-excess
	Shock developed	Shock developed	Shock developed
	Coma developed	Coma developed	Coma developed
	Seizures developed	Treatment	Treatment
	Sepsis developed		
	Treatment		

Abbreviations: CSH, cause-specific hazard; SDH, subdistribution hazard.

Artesunate had a wider range of time to death (0.5–30 days) than quinine (0.5–17 days), and significantly more patients in the artesunate arm died after 7 days (8.5%) than in the quinine arm (2.0%; *P* = .019). Artesunate significantly decreased the rate and cumulative incidence of death (Figure 4), with adjusted CSH and SDH ratios both 0.60 (Table 3).

## DISCUSSION

LOS is important in planning healthcare delivery [7], with strategies to reduce LOS proving effective in alleviating pressure on bed capacity [13]. Patients who die or who are discharged both contribute to bed occupancy and resource use. Thus, both metrics are important for planning, and a competing-risk approach, accounting for both, produces estimates relevant to real-world service planning [7].

In contexts similar to this study, a median LOS (time to discharge or death in-hospital) of 5 days, a median time to discharge of 6 days, and a mean time to death of 2.5 days (the proportion of deaths was too low to calculate a median) can be expected. Planners can also expect most severe malaria patients to be discharged and most deaths to occur within the first week, demonstrated by the cumulative incidence pattern (Figure 2), which provides data along with expected LOS to anticipate bed availability, the timing and quantity of resources required, and to project the budget needed to manage expected cases [9].

These predictions need to account for factors that influence LOS [9]. As expected, a lower cumulative incidence of discharge and higher incidence of death were found in sicker patients; individuals with lower GCS, renal dysfunction (BUN >21 mg/dL), and acidosis (base-excess <−3). In addition to the development of anemia, patients who developed clinical complications also had longer LOS and reduced incidence of discharge, and, in contrast with the overall sample, most were discharged after 7 days. These insights offer targets for screening and management of conditions to reduce LOS and relieve pressure on the need for hospital beds [13].

Artesunate decreases mortality compared to quinine [18], and a greater proportion of patients in the artesunate arm died after 7 days, with a wider range of outliers. It could be reasoned that artesunate prolonged time to death of patients who would otherwise have died earlier. It could also be inferred that patients who might have died had they received quinine but survived because of artesunate’s mortality benefits may be sicker than other patients who survived to discharge, taking longer to recuperate.

The median time to discharge was 6 days for both treatment arms. However, median times on their own can be misleading, only describing 1 time point and offering no information about event distribution [31]. Cumulative incidence illustrates the pattern of events, and exploration using a competing-risk approach revealed that artesunate increased the cumulative incidence of discharge compared to quinine by 8.8% (Table 3 and Figure 4).

This is an important consideration; changing to artesunate as first-line treatment should reduce mortality incidence, in turn, increasing the number of patients discharged. As severe malaria patients stay longer if they survive to discharge than if they die (Figure 3), this would lengthen overall LOS. Combined with the prolonged range of time to discharge in the artesunate arm, artesunate has the potential to significantly lengthen LOS in severe malaria patients.

Choice of first-line treatment is based on multiple factors, including impact on health outcomes, availability, ease of use, cost [32], and resource consumption, such as LOS [9]. While artesunate is justified as first-line treatment by improvements in health (reduced mortality [18] and increased cumulative incidence of discharge), the potential to increase overall LOS and subsequently the use of resources must be accounted for when planning services.

Only assessing time to discharge with conventional Cox regression means that the contribution of time to death on resource use would be overlooked and that systolic blood pressure, respiratory rate, base-excess, development of shock, and choice of treatment would not have been considered in service planning, as they were nonsignificant in the CSH model for discharge (Table 4). A less nuanced analysis results from not exploring time to death and the competing-risk model, leading to different conclusions on valuable predictors, such as artesunate’s influence on LOS. This could potentially influence the planning of strategies to reduce LOS and maximize resource efficiency.

Another limitation of using conventional survival analysis in the presence of competing events is overestimation of cumulative incidence. The Kaplan-Meier complement overestimated cumulative incidence of both discharge (100% compared to 80% estimated by the competing risk approach) and death (40% compared to 20% estimated by the competing risk approach). Added together, the sum of death and discharge occurring (the only 2 possible outcomes) is an impossible 140%, illustrating the lack of precision when competing events are not accounted for (Figure 2).

Competing events are found in many studies published in high-impact journals [33] and are usually inappropriately treated as censored observations [21]. Despite misunderstandings of the required Kaplan-Meier assumptions, a lack of awareness of the competing-risk approach and the historically poor availability of competing-risk software packages [34], competing-risk methods have been increasingly used to analyze noncommunicable diseases [22, 23, 35, 36]. However, studies that apply it to communicable-disease analysis are still limited.

CSH analysis reveals insights into etiological associations between variables and discharge [24]; however, these are based on a hypothetical world where other events do not take place. A proportion of malaria patients will die, influencing the cumulative incidence of discharge [33]. This makes SDH analysis, associated with cumulative incidence, particularly useful for planning in contexts where competing events exist [22]. It is imperative to explore both analyses to holistically understand the LOS in severe malaria.

There were limitations to our study. A number of the factors that affect malaria LOS found in the literature search (Supplementary Table 2) were not available in this dataset and thus not analyzed. Furthermore, post-treatment bias could be introduced by the variables that developed during hospitalization. To mitigate this, all analyses were adjusted for treatment as a fixed effect.

The sample was confined to the Asia-Pacific region, potentially limiting generalizability to Africa where 90% of cases occur [1]. At the same time, patients came from multiple sites across South-East Asia, resulting in large intercountry differences that could confound findings. Variation could be due to differences in clinical factors on admission (Supplementary Table 5) or explained by intrinsic differences in country disease profiles and services offered [2]. This was addressed by adjusting all analyses for the sample country as a fixed effect; however, the implication that LOS is affected by the setting should be considered when translating results to decision-making in a specific context.

## CONCLUSIONS

LOS in severe malaria is influenced by demographic, clinical, and treatment factors that, along with cumulative incidence and expected time to both discharge and death, should be incorporated into planning to improve service efficiency. These metrics are also useful in decisions on treatment choice; artesunate increases cumulative incidence of discharge and decreases the cumulative incidence of death, reinforcing its recommendation as first-line treatment. However, its potential to increase overall LOS and the use of resources should be accounted for when planning malaria services.

Exploration of both CSH and SDH analyses can aid holistic understanding of variable relationships with LOS. Competing risks should be considered when designing and interpreting communicable-disease studies, as ignoring them leads to overestimation of cumulative incidence and misrepresentation of variable associations, LOS, and the pattern of discharge and death over time. This, in turn, can lead to disparate conclusions, which have important implications for the policy decisions that impact the planning of malaria service delivery.

## Supplementary Data

Supplementary materials are available at *Clinical Infectious Diseases* online. Consisting of data provided by the authors to benefit the reader, the posted materials are not copyedited and are the sole responsibility of the authors, so questions or comments should be addressed to the corresponding author.

Supplementary Appendix

## Notes

***Acknowledgments.*** We thank the South-East Asian Quinine Artesunate Malaria Trial group (members listed in the Supplementary Materials) and staff at the Mahidol-Oxford Research Unit for their help, support, and advice. We thank Cancer Research UK and the University of Oxford for the grant to E. O. O. to cover publication-related fees.

***Financial support.*** The initial analysis was completed to fulfill the thesis requirements for the Masters in International Health and Tropical Medicine at the University of Oxford, undertaken by C. M. K. in 2016–2017, which was supported by a joint scholarship from the Skye Foundation and the Weidenfeld-Hoffman Trust.

***Potential conflicts of interest.*** All authors: No reported conflicts of interest. All authors have submitted the ICMJE Form for Disclosure of Potential Conflicts of Interest. Conflicts that the editors consider relevant to the content of the manuscript have been disclosed.
