# Supplementary material for: A Competing-Risk Approach for Modeling Length of Stay in Severe Malaria Patients in South-East Asia and the Implications for Planning of Hospital Services
Source: Clin Infect Dis. 2018 Mar 19;67(7):1053–62. doi: 10.1093/cid/ciy211 (PMC6137114; doi:10.1093/cid/ciy211)
Supplement: Supplementary Appendix [file ciy211_suppl_supplementary_appendix.docx]

**Appendix**

- Table A1. Search strategy for literature review of variables associated with LOS in malaria
- Table A2. Factors affecting LOS or the requirement for intensive care in malaria patients, found in a literature review, and their availability in the SEAQUAMAT dataset
- Table A3. Univariable analysis for time to discharge, using a conventional Cox regression model to obtain a cause-specific hazard ratio, and a Fine and Gray competing-risks method to obtain a subdistribution hazard ratio, with fixed adjustment for country and treatment arm
- Table A4. Univariable analysis for time to death, using a conventional Cox regression model to obtain a cause-specific hazard ratio, and a Fine and Gray competing-risks method to obtain a subdistribution hazard ratio, with fixed adjustment for country and treatment arm
- Table A5. Characteristics of the adult population of the SEAQUAMAT dataset by sample country
- SEAQUAMAT group list

| **Database searched** | **Terms** |
| --- | --- |
| MeSH database on PubMed | "Length of Stay"[Major] AND "Malaria"[Mesh]  1 result specific to Goa (Vel*ip et al*>, 2006) |
|  | "Length of Stay"[Mesh] AND "Malaria"[Major]  45 results – 18 relevant  Irrelevant: outcome: time to death, time to medication administration or occurrence of death, bed net burns, incubation period, time to presentation, and complications. A number were small case series descriptions and duplicated papers. One was a randomised controlled trial of a diagnostic test. Two papers only focussed on vivax malaria. |
| Cochrane Library | Malaria and length of stay  1 irrelevant result on n-acetylcysteine for sepsis |
| Google Scholar | malaria AND length of stay OR prognostic OR intensive care  71 900 results – titles scanned for relevance to supplement above searches.  28 relevant |

*Table A1. Search strategy for literature review of variables associated with LOS in malaria*

| **Factor** | **Available in SEAQUAMAT dataset** |
| --- | --- |
| **Demographics** | |
| Age | Yes |
| Gender | Yes |
| Country/hospital site | Yes |
| Distance from hospital | No |
| Season | No |
| Delay in presentation | No |
| Referral | No |
| **Clinical factors** | |
| Comorbidities | No |
| Low blood pressure | Yes |
| Shock | Yes |
| Respiratory distress | Yes |
| Hepatitis | No |
| Coma | Yes |
| Fever | Yes |
| Haemoglobinuria | No |
| Renal failure | Yes |
| Seizures | Yes |
| Outcome | Yes |
| **Laboratory results** | |
| Leucocytosis | No |
| Thrombocytopaenia | No |
| Type of malaria | No – all had *P. falciparum* |
| Hyperparasitaemia | Yes |
| Schizontaemia | No |
| **Treatment** | |
| Antimalarials | Yes |
| Vitamin A | No |

*Table A2. Factors affecting LOS or the requirement for intensive care in malaria patients, found in a literature review, and their availability in the SEAQUAMAT dataset*

| **Covariate** | **Cause-specific hazard (rate of discharge)** | | **Subdistribution hazard (cumulative incidence of discharge)** | |
| --- | --- | --- | --- | --- |
|  | **CSH ratio** | **p value** | **SDH ratio** | **p value** |
| **Demographics** | | | | |
| Country  Reference (Myanmar)  2 (Bangladesh)  3 (India)  4 (Indonesia) | 1.44 (1.24-1.68)  1.38 (1.11-1.71)  2.02 (1.68-2.42) | **<0.001**  **0.004**  **<0.001** | 0.96 (0.83-1.11)  1.03 (0.84-1.27)  1.73 (1.44-2.07) | 0.577  0.760  **<0.001** |
| Age | 0.99 (0.99-1.00) | 0.297 | 0.99 (0.99-1.00) | **<0.001** |
| Gender  Reference (female)  1 (Male) | 0.92 (0.80-1.07) | 0.297 | 0.98 (0.85-1.13) | 0.748 |
| **Clinical factors on admission** | | | | |
| Systolic blood pressure | 0.99 (0.99-1.00) | 0.455 | 0.99 (0.99-1.00) | **0.007** |
| Respiratory rate | 1.00 (0.99-1.01) | 0.954 | 0.99 (0.98-0.99) | **0.001** |
| Temperature | 1.09 (1.03-1.15) | **0.001** | 1.12 (1.06-1.18) | **<0.001** |
| Glasgow coma scale | 1.05 (1.04-1.07) | **<0.001** | 1.13 (1.11-1.15) | **<0.001** |
| Seizures  Reference (absent)  1 (present) | 0.84 (0.67-1.05) | 0.133 | 0.77 (0.63-0.85) | **0.015** |
| **Laboratory results on admission** | | | | |
| Blood urea nitrogen | 0.99 (0.99-0.99) | **<0.001** | 0.99 (0.98-0.99) | **<0.001** |
| Base-excess | 1.03 (1.02-1.04) | **<0.001** | 1.07 (1.06-1.09) | **<0.001** |
| Log parasite count | 0.95 (0.93-0.98) | **<0.001** | 0.93 (0.91-0.95) | **<0.001** |
| Haemoglobin | 1.02 (1.00-1.04) | **0.049** | 1.02 (1.00-1.03) | 0.087 |
| **Clinical conditions developed during admission** | | | | |
| Shock  Reference (absent)  1 (present) | 0.35 (0.19-0.63) | **<0.001** | 0.13 (0.07-0.22) | **<0.001** |
| Coma  Reference (absent)  1 (present) | 0.50 (0.37-0.68) | **<0.001** | 0.28 (0.21-0.37) | **<0.001** |
| Seizures  Reference (absent)  1 (present) | 0.40 (0.26-0.60) | **<0.001** | 0.27 (0.19-0.40) | **<0.001** |
| Sepsis  Reference (absent)  1 (present) | 0.35 (0.26-0.46) | **<0.001** | 0.37 (0.28-0.48) | **<0.001** |
| Anaemia  Reference (absent)  1 (present) | 0.67 (0.44-1.02) | 0.059 | 0.81 (0.58-1.14) | 0.235 |
| **Treatment** | | | | |
| Previous effective antimalarials  Reference (no treatment): n (%)  1 (previous treatment): n (%) | 0.87 (0.74-1.03) | 0.101 | 0.90 (0.77-1.04) | 0.159 |
| Treatment arm  Reference (quinine)  1 (artesunate) | *Table A3. Univariable analysis for time to discharge, using a conventional Cox regression model to obtain a cause-specific hazard ratio, and a Fine and Gray competing-risks method to obtain a subdistribution-hazard ratio, with fixed adjustment for country and treatment arm*  1.04 (0.92-1.18) | 0.559 | 1.25 (1.11-1.41) | **<0.001** |

| **Covariate** | **Cause-specific hazard (rate of death)** | | **Subdistribution-hazard (cumulative incidence of death)** | |
| --- | --- | --- | --- | --- |
|  | **CSH ratio** | **p value** | **SDH ratio** | **p value** |
| **Demographics** | | | | |
| Country  Reference (Myanmar)  2 (Bangladesh)  3 (India)  4 (Indonesia) | 2.06 (1.53-2.78)  1.65 (1.08-2.50)  0.83 (0.51-1.24) | **<0.001**  **0.019**  0.440 | 1.90 (1.42-2.54)  1.54 (1.03-2.31)  0.72 (0.45-1.15) | **<0.001**  **0.037**  0.164 |
| Age | 1.02 (1.01-1.03) | **<0.001** | 1.02 (1.01-1.03) | **<0.001** |
| Gender  Reference (female)  1 (Male) | 0.96 (0.71-1.29) | 0.790 | 0.97 (0.72-1.30) | 0.831 |
| **Clinical factors on admission** | | | | |
| Systolic blood pressure | 1.01 (1.00-1.02) | **0.007** | 1.01 (1.00-1.02) | **0.011** |
| Respiratory rate | 1.03 (1.02-1.04) | **<0.001** | 1.03 (1.02-1.04) | **<0.001** |
| Temperature | 0.89 (0.79-0.99) | **0.040** | 0.87 (0.78-0.97) | **0.013** |
| Glasgow coma scale | 0.81 (0.79-0.84) | **<0.001** | 0.81 (0.78-0.83) | **<0.001** |
| Seizures  Reference (absent)  1 (present) | 1.35 (0.91-1.99) | 0.132 | 1.38 (0.95-2.01) | 0.090 |
| **Laboratory results on admission** | | | | |
| Blood urea nitrogen | 1.01 (1.01-1.02) | **<0.001** | 1.01 (1.01-1.02) | **<0.001** |
| Base-excess | 0.87 (0.86-0.89) | **<0.001** | 0.87 (0.85-0.89) | **<0.001** |
| Log parasite count | 1.19 (1.12-1.28) | **<0.001** | 1.21 (1.11-1.30) | **<0.001** |
| Haemoglobin | 0.99 (0.95-1.03) | 0.713 | 0.99 (0.95-1.03) | 0.538 |
| **Clinical conditions developed during admission** | | | | |
| Shock  Reference (absent)  1 (present) | 5.86 (4.20-8.18) | **<0.001** | 6.44 (4.91-8.43) | **<0.001** |
| Coma  Reference (absent)  1 (present) | 4.06 (3.03-5.45) | **<0.001** | 4.41 (3.38-5.76) | **<0.001** |
| Seizures  Reference (absent)  1 (present) | 3.26 (2.27-4.68) | **<0.001** | 3.70 (2.69-5.10) | **<0.001** |
| Sepsis  Reference (absent)  1 (present) | 2.19 (1.52-3.17) | **<0.001** | 2.59 (1.86-3.60) | **<0.001** |
| Anaemia  Reference (absent)  1 (present) | 0.93 (0.41-2.10) | 0.867 | 1.02 (0.47-2.24) | 0.956 |
| **Treatment** | | | | |
| Previous effective antimalarials  Reference (no treatment): n (%)  1 (previous treatment): n (%) | 1.05 (0.76-1.46) | 0.755 | 1.07 (0.78-1.47) | 0.657 |
| Treatment arm  Reference (quinine)  1 (artesunate)  *Table A4. Univariable analysis for time to death, using a conventional Cox regression model to obtain a cause-specific hazard ratio, and a Fine and Gray competing-risks method to obtain a subdistribution-hazard* ratio, *with fixed adjustment for country and treatment arm* | 0.62 (0.48-0.81) | **<0.001** | 0.61 (0.48-0.79) | **<0.001** |

| **Variable** | **Myanmar** | **Bangladesh** | **India** | **Indonesia** | **p value** |
| --- | --- | --- | --- | --- | --- |
| **Demographics** | | | | | |
| Age in years (median and IQR) | 26(21;40) | 30(22;40) | 31(22;41) | 26(21;32) | **0.003** |
| Gender  Reference (females): (%)  1 (males): (%) | 22.8%  77.3% | 19.6%  80.4% | 19.0%  81.0% | 39.8%  60.2% | **<0.001** |
| **Clinical factors on admission** | | | | | |
| Systolic blood pressure in mmHg (median and IQR) | 100(90;112.5) | 110(100;120) | 120(110;130) | 100(85.5;110) | **<0.001** |
| Respiratory rate in breaths per minute (median and IQR) | 28(22;33) | 22(20;28) | 24(20;28) | 32(24.5;40) | **<0.001** |
| Temperature in C (mean and SD) | 38.3 (1.1) | 38.0 (1.1) | 37.8 (1.2) | 37.6 (1.3) | **<0.001** |
| Glasgow Coma Scale score (median and IQR) | 12(9;15) | 11(8;15) | 10(7;15) | 15(10.5;15) | **<0.001** |
| Seizures  Reference (absent): (%)  1 (present): (%) | 91.0%  9.0% | 92.3%  7.6% | 75.9%  24.1% | 96.4%  3.6% | **<0.001** |
| **Laboratory results on admission** | | | | | |
| Blood urea nitrogen mg/dL (median and IQR) | 27(15;55) | 30(18;59) | 32.5(19;64.5) | 31.74(19;66) | 0.153 |
| Base-excess mmol/L (mean and SD) | -3.5 (7.2) | -5.2 (6.8) | -5.1 (8.0) | -4.5 (5.6) | **0.008** |
| Log parasite count (mean and SD) | 10.5 (2.4) | 10.6 (2.4) | 10.7 (2.7) | 10.6 (2.2) | 0.661 |
| Haemoglobin mg/dL (mean and SD) | 10.0 (3.5) | 10.5 (3.1) | 11.0 (3.4) | 9.5 (3.3) | **<0.001** |
| **Clinical conditions developed during admission** | | | | | |
| Shock  Reference (absent): (%)  1 (present): (%) | 96.1%  3,9% | 95.7%  4.3% | 93.4%  6.6% | 95.4%  4.6% | 0.602 |
| Coma  Reference (absent): (%)  1 (present): (%) | 89.3%  10.7% | 96.7%  3.3% | 81.0%  19.0% | 93.4%  6.6% | **<0.001** |
| Seizures  Reference (absent): (%)  1 (present): (%) | 95.1%  4.9% | 95.0%  5.0% | 96.4%  3.6% | 95.4%  4.6% | 0.923 |
| Sepsis  Reference (absent): (%)  1 (present): (%) | 96.4%  3.6% | 95.0%  5.0% | 92.0%  8.0% | 81.6%  18.4% | **<0.001** |
| Anaemia  Reference (absent): (%)  1 (present): (%) | 96.1%  3.9% | 98.6%  1.4% | 98.5%  1.5% | 98.5%  1.5% | 0.068 |
| **Treatment** | | | | | |
| Prior effective malaria treatment  Reference (absent): (%)  1 (present): (%) | 83.1%  16.9% | 84.7%  15.3% | 70.1%  29.9% | 83.7%  16.3% | **0.001** |
| Study treatment  Reference: (quinine): (%)  1 (artesunate): (%) | 48.9%  51.1% | 51.2%  48.8% | 50.4%  49.6% | 49.0%  51.0% | 0.911 |
| **Outcome** | | | | | |
| Outcome  Reference (survived): (%)  1(died): (%)  n= number IQR = Inter-quartile range SD= Standard Deviation  *Table A5. Characteristics of the adult population of the SEAQUAMAT dataset by sample country* | 84.8%  15.2% | 72.3%  27.8% | 76.6%  23.4% | 88.8%  11.2% | **<0.001** |

SEAQUAMAT group list

*Bangladesh*

Prof M Abul Faiz, Emran Bin Yunus, M Ridwanur Rahman, Prof Faridul Islam, Prof M Gofranul Hoque, Mahatab Uddin Hasan, Rasheda Samad, and research assistants and study nurses.

*Myanmar*

Soe Aung, Soe Thein, Prof Marlar Than, Prof Ye Thwe, Prof Khin Mae Ohn, San Hla, Saw Lwin, Ye Htut, Khin Lin, Myat Phone Kyaw, Ne Win, Win Ne Aung, Myint Win, Aung Zaw Oo, Zaw Aung, Ohnmar Myint Shein, Mar Kyi, Win Myint, Khin Phyu Pyar, Kyaw Nyein, and Kyu Win. Coordinated by the Ministry of Health, Union of Myanmar, and the Department of Medical Research, Yangon.

*India*

Saroj K Mishra, Sanjib Mohanty, Rajya Bardhan Pattnaik, Sanjay K Acharya, Anita Mohanty, and Devendranath Mohapatra.

*Indonesia*

Emiliana Tijtra, Prof Nicholas Anstey, Ric Price, Tjandra Handoyo, Dekrit Gampamola, Enny Kenangalem, Denny Takaendengan, Hardiyanto, Ardi Lampat, and Paul Harijanto. Coordinated by the National Institute of Health Research and Development, Ministry of Health, Republic of Indonesia, and the Menzies School of Health Research and Charles Darwin University, Darwin, Australia.

*Clinical, laboratory, statistical, and logistic support*

Wellcome Unit and Shoklo Malaria Research Unit, Faculty of Tropical Medicine, Mahidol University, Bangkok, Thailand: Arjen Dondorp, François Nosten, Nick Day, Kasia Stepniewska, Prakaykaew Tipmanee, Sam Douthwaite, Kamolrat Silamut, and Stephane Proux.

*Coordinating committee*

Arjen Dondorp, François Nosten, Nick Day, Kanchana Pongsawat, and Prof Nick White (chair).

*Writing committee*

Arjen Dondorp, François Nosten, Kasia Stepniewska, Nick Day, and Prof Nick White.

*Data and safety monitoring committee*

David Lalloo, Sarah Walker, and Prof Tim Peto (chair).
